# Using interviews and observations in clinical practice to enhance authenticity in virtual patients for interprofessional education

**DOI:** 10.1186/s12909-020-02379-9

**Published:** 2020-11-25

**Authors:** Desiree Wiegleb Edström, Niklas Karlsson, Samuel Edelbring

**Affiliations:** 1 School of Medical Sciences, Örebro University, Örebro, Sweden; 2 Department of Medicine, Solna, Karolinska Institutet, Stockholm, Sweden; 3 School of Health Sciences, Örebro University, Örebro, Sweden

**Keywords:** Virtual patients, E-learning, Interprofessional education

## Abstract

**Background:**

Interprofessional collaboration is increasingly important in healthcare, but interprofessional education (IPE) faces challenges, such as different study programmes with varied schedules and campuses. These challenges can be met, in part, by using web-based virtual patients (VPs) as a tool in IPE. However, demands for relevant patient presentations and clinical practice increase when VPs are used by students from different programmes. The aim of this study was to improve the presentation of professional perspectives regarding nurses and physicians and their collaboration in order to increase the clinical authenticity of existing VPs.

**Methods:**

Clinical observations were conducted to gain familiarity with the context. Semi-structured interviews were performed with individual nurses and physicians with experience of patients with leg ulcers. The interviews were recorded, transcribed and analysed using thematic analysis.

**Results:**

The clinical observations exposed a lack of interprofessional collaboration in practice with regard to patients with leg ulcers**.** The interview analysis resulted in two themes: Clinical care and Organizational structure. The theme Clinical care included nursing with a holistic approach to the patient and awareness of the patient’s well-being, including nutrition and home situation. The theme Organizational structure revealed a lack of teamwork in primary care. The interviewees stressed learning together and sharing responsibility, and they emphasised the importance of implementing interprofessional learning in the education of nurses and physicians in order to stimulate future teamwork. The VP should offer a broad medical history so that healthcare students are made aware of how a disease can affect the patient’s social situation, and thereby illustrate the importance of interprofessional collaboration. The information should also be comprehensive and clear, leading to a diagnosis, so the student can gain clinical knowledge and build a foundation for discussion of treatment.

**Conclusions:**

Interviews and observations in clinical practice can be used to enhance authenticity in VPs for interprofessional learning. A thorough look at authentic clinical environments can enrich and improve educational settings using VPs, and it can highlight the challenges students can encounter in clinical care of the patient and in an organisation with regard to interprofessional collaboration.

## Background

Interprofessional collaboration is important in healthcare, which is becoming increasingly complex due to an ageing population and more advanced medical care, thereby requiring contributions from different areas of expertise in a collaborative manner [1]. Consequently, there is a growing need for interprofessional learning activities. Interprofessional learning occurs when students from two or more professions learn about, from, and with each other to enable effective collaboration and improve health outcomes [2]. It has been shown that students’ perceptions and attitudes towards interprofessional collaboration can be enhanced through IPE [3]. The increased need for IPE is often addressed via simulation-based learning to foster collaborative practice [4]. However, IPE faces challenges because of study programmes with diverse schedules and students being located across different campuses. Online simulations can bridge the geographical gaps between campuses and offer time flexibility, thus presenting interesting add-ons to campus-based IPE.

Clinical cases can be presented in a student-interactive manner through virtual patients (VPs). A VP is “a specific type of computer-based program that simulates real-life clinical scenarios; learners emulate the roles of healthcare providers to obtain a history, conduct a physical exam, and make diagnostic and therapeutic decisions” [5]. VP scenarios can be designed for independent student access regardless of profession, schedule, or access to campus rooms. VPs have been used to address IPE objectives in providing awareness of one’s own roles and responsibilities, and those of other professions [6]. The simulation media present limitations to case authenticity, such as with paper-based static patient presentations in which all patient information is limited and presented simultaneously and, thus, have been found to present a fragmented picture and be reductionist in nature [7]. The computerized modality of VPs offers a way to present rich patient information in addition to only presenting complaint, salient symptoms and lab data. However, a problem with simulation-based clinical presentations could be that scenarios authored by teachers risk to be coloured by their professional and personal perspectives and present scenarios that do not align with current clinical practice and current illness panoramas [8].

Bateman and colleagues argue that real-life clinical properties should be incorporated into VPs for student learning [9]. Bateman and co-workers conducted a grounded theory study of the influence of VP design on undergraduate medical students. They found four categories relating to VP design: VP Constructions, External Preconditions, Student-VP Interaction and Consequences. One of the sub-categories of VP Construction was “Clinical properties,” which referred to how the VP cases related to actual clinical practice [9]. Furthermore, the level of difficulty in VPs should be relevant to the student group [10], and the educational design should foster clinical reasoning [11]. Because of clinical reasoning being a multi-faceted process, the VPs should describe authentic clinical practices and be rich in their patient presentation making possible for students to see and reflect on different aspects. In IPE it is important that clinical presentations reflect various professional perspectives, providing fruitful learning situations for several professions. Bateman and colleagues outline four categories relating to VP design and their influence on medical students’ learning. The issue of aligning educational patient presentations with population data has also been raised, pointing towards the need to reflect real-life patients and practices [8]. It is therefore important to improve VP design, which can be performed by enhancing the VPs’ authenticity regarding clinical practice for different health professionals. However, no protocol or standard to better align VPs with real-life clinical authenticity has yet been established. We therefore explored ways of enhancing authenticity by conducting observations and interviews in relation to two existing VP cases.

### Aim

Our aim was to increase the knowledge of how to enhance the authenticity of authored clinical cases in the context of interprofessional clinical reasoning.

## Methods

We used observations from clinical professional practice and interviews with health professionals to enrich and enhance the authenticity of two existing VP cases.

### Virtual patients

The VP scenarios are two patients with leg ulcers visiting a primary care, one with a venous leg ulcer and one with an arterial leg ulcer. In light of the observations and interviews, we examined our two web-based VPs that had already been designed with reference to clinical practice in and professionals’ perspectives on IPE. The VP scenarios are two patients with leg ulcers visiting a primary care, one with a venous leg ulcer and one with an arterial leg ulcer. We selected leg ulcer diagnosis as the topic for our VPs because it depends on teamwork to be successfully treated [12], thus illustrating the importance of interprofessional collaborative practice. The web-based Virtual Interactive Case platform [13], which allows free navigation in gathering patient data in a linear problem-solving mode [14], was used. Data and actions are structured in different sections, such as medical history, medical examination, medical investigations (X-ray, laboratory results, etc.) and medical reports. The students can evaluate images, such as photos of the leg ulcers, and different scales and measurements for nutrition, pain, peripheral circulation, and others. The final step is to suggest a plausible diagnosis and suitable therapies, after which the student can access built-in feedback.

The VP cases were designed by a physician (DWE), together with a nurse who has experience designing VPs and is involved in VP research. Nursing expertise was further gathered by consulting a nurse with experience of leg ulcers who is also an educator. We choice leg ulcers as the conditions involve several health care professions and students from several healthcare programmes. It is, therefore, a well-functioning topic for IPE, illustrating the importance of interprofessional collaboration.

### Clinical observations

Clinical observations were performed by the researcher NK at two sites: a primary care setting and a hospital dermatology unit. A nurse or a physician treating patients with leg ulcers was shadowed and observed. The purpose was to acquire an understanding of the nurse’s and physician’s roles and gain familiarity with the context. A logbook on the interprofessional collaboration was kept. Ten patients were observed. One nurse was shadowed at the primary care setting for two morning shifts. At the hospital, one nurse was shadowed for one morning shift, and one physician for another morning shift.

### Interviews

In total, five persons were interviewed, three nurses and two physicians who were recruited through either the primary care director or the hospital department director, based on the criterion of experience of treating leg ulcers. One general practitioner (GP) and one nurse working in primary care participated. Two nurses from a dermatology hospital ward, one of whom has also lectured on leg ulcers, and one dermatologist from the same hospital, who has treated patients with leg ulcers and given lectures on the subject, took part. The interviews were semi-structured, and each participant was interviewed separately. The interview guide included questions regarding professional clinical leg ulcer practice, what should constitute a leg ulcer VP case, and what the participants considered important for enabling interprofessional collaboration.

The interviews were performed at the workplace, with one interview via phone. Each interview was audio recorded and lasted 15–30 min. The observation and interview data are stored in a dedicated space with the main researcher (DWE). The audio files therein are stored on digital memory cards and the content is anonymous and do not contain personal identifiable information.

### Data analysis

The clinical observations logbook was analysed to gain knowledge regarding the daily work with leg ulcers at the primary care setting and at the hospital. Material relevant to the study’s aim was analysed and synthesised into a short description.

The interviews were audio recorded, transcribed and read repeatedly in order to become familiar with the data. Analyses were performed by thematic analysis [15]. Each interview was first analysed separately, then horizontally throughout all interviews. The words and sentences in the transcript were coded in relation to clinical leg ulcer practice, interprofessional collaboration and VP case presentation. The initial codes were identified collaboratively and then interpreted and worked into themes. The analysis was repeated in a process of defining and further refining until the final themes were established. The primary phase of the analysis and creation of themes was performed by authors DWE and NK. Author SE was involved in the second stage of critically scrutinising the interpretations. The three authors have different backgrounds: a medical student who previously studied nursing (NK), a lecturer and senior consultant in dermatology (DWE) and a healthcare profession educator and researcher (SE). This increased the probability of understanding and interpreting the data in many ways, acknowledging multiple realities [16]. The study was approved by the regional ethical committee in Stockholm (Dnr: 2014/615–31/1).

## Results

### Observations in clinical practice

The observation in clinical practice provided an overall understanding of everyday clinical practice regarding leg ulcers. In our setting the observations revealed differences in practice between the primary care and hospital settings. At the primary care setting, a nurse working independently with the patients carried out the wound-related work. There was no collaboration with a physician, such as a GP. A GP appointment was only made if the nurse considered it necessary, for example if the nurse needed advice because something was unclear, if the ulcer was getting worse or if the patient needed special shoes. There was no time for collaboration with the GP to discuss the patients, the different wound dressings or the underlying causes of or diagnosis of the leg ulcer. A few of the patients who were treated by the nurse had been examined by a physician regarding the leg ulcer. Patients with leg ulcers were not placed on the GP’s regular schedule. The patients were treated by a nurse and the physician was only occasionally involved in the treatment, which is common practice in the Swedish context of treating leg ulcers in primary care [17]. Therefore, there was no observation of a GP in the primary care setting to perform.

In the hospital setting, however, the physician initially met the patients to take a medical history and perform a physical examination, resulting in a preliminary diagnosis. The physician and nurse collaborated on the leg ulcer management and discussion of the wound dressing. They also planned together follow-up visits to the nurse at the primary care setting and the nurse in the dermatology department. The nurse in the dermatology department also scheduled patients on her own at the follow-up visit, working independently. There was interprofessional collaboration at the hospital, but no interprofessional encounters were observed in the primary care setting.

### Interviews

The interview analysis resulted in two themes: Clinical care and Organizational structure (see Table 1).

**Table 1:** Codes and themes from the interviews

Analytic codes	Sub-themes	Themes
Holistic approach	Nursing	Clinical care
Nutrition		
Medical history	Initial handling	
Investigation		
Referrals		
Documentation	Action	
Diagnosis		
Treatment		
Information to the patient		
Follow-up		
Lack of cooperation	Lack of teamwork	Organizational structure
Lack of continuity		
Interest in other professions	Learning	
Learning together		
Shared responsibility	Responsibility	
Clear roles		

### Clinical care

The theme Clinical care comprised the sub-themes *Nursing*, *Initial handling* and *Action*.

*Nursing* involved a Holistic approach and good Nutrition. The holistic approach included an extensive medical history to identify the patient’s main problems, the entirety of the patient’s situation, including their home circumstances, need for aid and facilities, and the patient’s treatment expectations. It was important that the patient received appropriate help in order to continue their normal daily life.

*I believe it is very important to know the medical history in order to know what the main problem is, why the patient has this ulcer.* (Physician)

Nutrition included weight, such as being over- and underweight, and a nutritious diet which affected the patient’s general health, including secondary diseases such as diabetes. The data included questions about patients’ food intake with the ambition of achieving well-balanced nutrition with an impact on the healing process.

*If you don’t eat a balanced diet, with proteins, vitamins and minerals, the healing of the ulcer will be influenced*. (Nurse)

The sub-theme *Initial handling* included Medical history, Investigation, and Referrals. The interviewers highlighted that Medical history should not only focus on the VP’s specific diagnosis*,* but also cover whether the patient has other diseases or an underlying disease which might affect the leg ulcer, and what medications are prescribed.

*… it is important to know how long the patient has had leg ulcers, how they appeared, and which diseases the patient has …* (Physician)

Investigation emphasised the importance to get a more general picture of the patient’s health status including the leg ulcer. Investigation includes taking blood pressure, auscultating the heart, and conducting laboratory tests, e.g. blood tests for anaemia and kidney function. This information can have an impact on the prognosis, contribute to different proposals on the aetiology, and influence the treatment and care. Students have to also learn the clinical signs and symptoms that can cause and worsen the diagnosis. The main aim of investigating the leg ulcer is to learn to look for these signs and to further steer the investigation process and appropriate treatment. Such investigations include, for example, evaluating the blood circulation in the leg, and determining the pain from the ulcer or whether there is an ongoing infection. The interview participants pointed out the importance of clear and comprehensive information so the student can gain knowledge and stimulate clinical reasoning.

*… I want to know what kind of wound I am treating; I want to know the cause of the wound so I know what kind of treatment to choose …* (Nurse)

*… why is the patient in more pain, can it be an ongoing infection, or has something happened regarding the circulation …*? (Nurse)

Referrals and the possibility of referring the patient to an orthopaedist, physiotherapist, occupational therapist or podiatrist to help with different medical resources to improve the patient’s daily life were discussed in the interviews. Referrals could also be made for further investigation. In our two VPs referrals could be made for further investigation of the leg’s venous and arterial circulation to determine the aetiology and begin a suitable treatment approach.

The sub-theme *Action* included the following codes: Documentation, Diagnosis, Treatment, Information to the patient and Follow-up. Documentation of the patient’s healthcare is an important part of the nurse’s assignment. In our scenario it included documentation to describe the leg ulcer in relation to signs of healing or worsening, signs of infection and pain, the topical treatment used and the treatment outcome. The nurse writes the documentation in the medical report for both the nurse and the physician to follow the healing process.

*… to describe it [the ulcer] accurately, we measure it, take photos, and make sure there is time for these things in order to describe the ulcer correctly...* (Nurse)

The interviewees emphasised the importance of the VPs having a common and distinct Diagnosis with typical clinical signs. The students should be familiar with the most common leg ulcers and how to make a correct diagnosis. This is essential knowledge before choosing the treatment and management. This also implies clear and descriptive VP cases that are easy to interpret and understand.

*… if you don’t know the most common [diagnosis], then you cannot recognise when something comes that differs from the usual, which in many cases might be more dangerous or alarming … (Physician)*

Correct wound dressing and compression are the two main components of leg ulcer treatment [18]. The interviewees emphasised the importance of having basic knowledge of dressings and how they are expected to function and be used correctly. Compression is often a key factor in healing leg ulcers, especially venous leg ulcers, although it can only be applied if the blood circulation in the foot is normal or near normal.

*Yes, I believe that one should have some focus on which wound dressing is used, and have knowledge on what function the dressing has …* (Nurse)

Information to the patient should stimulate patients to become more involved in their treatment and thereby encourage them to take responsibility for their own health. The information could include motivation to use compression, to follow prescriptions, to visit the nurse regularly for changing the dressing, to change or improve their life habits such as nutrition or smoking, and to increase physical activities. Follow-up covers how the treatment has functioned, whether the patient is in less pain, and the need to involve other health professionals to optimise the wound healing. The students have to not only learn and be aware of the importance of follow-up for the patient, but also receive feedback on their own decisions.

*… you always evaluate the treatment, the dressing that has been used, whether it was good, if it was too wet, or too dry, or if it was stuck [in the wound] or it hurt.* (Nurse)

### Organizational structure

The theme Organizational structure included the sub-themes *Lack of teamwork* and *Learning* and *Responsibility*. 

The sub-theme *Lack of teamwork* concerned lack of cooperation, which is the absence of interaction between physicians and nurses in the treatment of leg ulcers in primary care. The nurse at the primary care setting had no collaboration with the GP and felt a lack of interest from the GPs regarding leg ulcers. The nurse therefore only involved the GP when the treatment was failing.

*...it is often like two different worlds when you are talking with the primary care centre; they [the nurses] do their work and the GPs their work, and only when the treatment fails do they call for the doctor.* (Nurse)

Lack of continuity refers to different physicians are managing the patient’s illness. The nurses experienced how the GPs considered leg ulcers to be the nurses’ task. This led to limited physician involvement. The nurses therefore perceived that they had the main responsibility for the patient.

*I believe the most common problem with collaboration is that there can be skilful nurses but doctors who are not familiar with leg ulcers. The nurses feel frustrated because they might not get the medical support they are asking for or need when it gets complicated...* (Physician)

The sub-theme *Learning* included the two codes Interest in other professions and Learning together*.* The importance of learning together and from each other emerged in the interviews. Education should include and stimulate awareness of and interest in other professions. The importance of being familiar with other professions and thereby also being aware of what to request and how each profession can take part in patient management was emphasised.

*...we have the same patients, we know what my role is, what a nurse can do, and it becomes much easier if you get information and education. I can then more easily accept collaboration, but if you don’t have a clue what a nurse, physiotherapist or an occupational therapist does … then I do not know how to utilise them either. (Physician)*

The sub-theme *Responsibility* included the two codes Shared responsibility and Clear roles*.* The interview participants discussed the importance of close collaboration between nurses and physicians in sharing the responsibility for improving the patient’s health and medical care. The nurse should have support from the doctor in their work, but the physician should also have backing from the nurse in keeping track of different wound dressings, for example.

*Thus, you can each contribute different things, so to be on the same level is fantastic, but I don’t think it is always that way. Everyone has their competence and together you can become rather good or at least hopefully good enough to solve the problem.* (Physician)

Clear roles involved the physician’s task to determine the diagnosis and the responsibility of writing an explicit prescription on how to treat the leg ulcer. The nurse’s role included caring for the patient, treatment and documentation.

*… it is important to have clear prescriptions from the doctor to the nurse on how the ulcer should be treated, that the doctor takes his/her responsibility on what dressing should be applied, how the wound should be treated, the need of a return visit,* etc. (Nurse)

The main result of the interviews was to show the importance of learning and using knowledge together. The VPs should contain the theme Clinical care with nursing, meaning a holistic approach to treatment and providing information to the patients that involves them in their own treatment. The interviews revealed the importance of clear and descriptive VPs with common diagnoses that are easy to interpret and understand. The VP should also include the theme Organizational structure not only regarding teamwork, learning together and shared responsibility, but also in emphasising the importance of clear roles.

### Consequences for virtual patient presentations

The VPs were adapted and enhanced using the observations and the interview results (Table 2). Insights from the fieldwork contributed to richer patient presentations, especially enhancing aspects of the caring perspective. The interviews provided comprehensive information on what a VP with a leg ulcer should include and how the approach differed between actors from the two involved professional perspectives. Viewing the VPs in light of the resulting two themes exposed weaknesses in our VP cases. It was particularly apparent that nursing aspects were lacking in our initial VP presentation. The anamnesis (the interview with the patient) and the medical history were enriched with more and explicit information from the VPs, by answering questions concerning their everyday situation and how the leg ulcer affected their daily life and well-being. More specific information about daily walks, exercises and sleeping habits was there for added. The VPs were further developed by including values for nutrition, such as mini-nutritional assessments. Taken together, these now enable a more holistic approach to the patient’s situation. Our results also showed the importance of characteristic VPs, identifying the main problem causing the patient’s disease. We therefore made the medical history more distinct and excluded details that might confuse the student, thereby facilitating clinical reasoning. Pain is common among these patients and was highlighted by the participants. The medical history was enriched by adding questions about pain. We also saw the importance of several alternatives of treatment combined with richer feedback. In our context wound dressings and explanations of their various functions were added to explicate the different kinds of wound dressing and how and when to apply them. General investigation was stressed as important and therefore electrocardiogram and pulmonary x-ray were added.

**Table 2:** Consequences for virtual patient presentation

Sub-themes	Analytic codes	Consequences for the VP
Nursing	Nutrition	We have included more values for nutrition such as mini-nutritional assessments
Initial handling	Medical history	The anamnesis (the interview with the patient) and the medical history were enriched with more and explicit information from the patients, answering questions concerning their everyday situation and well-being or pain from the leg ulcer.
Action	Treatment	Wound dressings and explanations of their various functions were added. Medical investigations were added as a part of general investigations.
Lack of teamwork	Lack of cooperation	The reports present documentation only from nurses at the primary care setting and nothing from a GP concerning the leg ulcer. Reflects current practice and are cause for students’ discussion.
Learning	Learning together	More distinct medical history and exclusion of details that might confuse the students
Responsibilities	Shared responsibility	We included more photos showing different situations of teamwork in order to make the VPs more realistic and illustrate the shared responsibility.

Table 2 showing relations between analysis and the consequences for our VPs

The theme Organizational structure, with the sub-themes Lack of teamwork and Responsibility, emerged in the interviews. The medical report of one of our VPs now only has information documented by nurses at the primary care setting and nothing from a GP concerning the leg ulcer, reflecting the physician’s lack of involvement, responsibility and teamwork. This illustrates the consequences of dysfunctional teamwork, resulting in a delayed diagnostic process and unnecessary pain and discomfort for the patient. The delay in the diagnosis of the VP’s leg ulcer helps the student gain an understanding of the consequences for patients when health professionals do not initially collaborate.

To further convey the clinical situation, we included more photos from clinical situations which depict activities such as meeting the patient, discussing with the patient, treating the leg ulcers, and teamwork, in order to make the VPs more realistic and illustrate the shared responsibility.

## Discussion

Our aim was to explore how to enhance the authenticity of a set of interprofessional VPs based on clinical practice. We found themes in the data which were then used in a process of increasing authenticity in existing VP cases.

Several changes were made as a result of our findings, which highlights the importance of considering information from several professions in VP authoring in an IPE setting. This information can be obtained by observation in clinical practice and by interviewing healthcare professionals in clinical practice. When using a VP presentation as the basis for an IPE activity, demands for multiple professional perspectives and richness in patient presentations arise. Students in health professions oriented towards caring and curing, should be able to relate to the way patients and the situations are portrayed and connect with selectable actions. Authored clinical scenarios risk being fragmented and losing their full educational potential because of limitations in modalities [7] or coloured by the authors’ professional and personal perspectives [8]. We therefore decided to examine our two already-designed VPs with reference to clinical practice and professionals’ perspectives in IPE.

The theme Clinical care emphasised the importance of nursing with a holistic approach to the patient. The VP cases were initially designed by a physician (DWE), thereafter incorporating a nursing perspective by inviting a nurse educator experienced in VP to the authoring. However, this initial approach was not sufficient for the nursing perspective to be salient in the VP presentations, and we had to enhance aspects of the caring perspective. Our results from interviewing nurses and physicians demonstrated the importance of common and illustrative diagnoses with distinct clinical information given to the student, providing good knowledge and understanding of the diagnosis and treatment as a foundation for further discussion and clinical reasoning. This is in alignment with, and meets a request from, a previous study performed with medical students, in which the students asked for relevant VPs, an appropriate level of difficulty, specific feedback, and help focussing on relevant learning points [10].

Our study showed lack of teamwork, with lack of involvement, continuity, and cooperation from the primary care physician. This is also expressed in similar settings, e.g. in a study with public health nurses in Norway that ascribed this lack of interprofessional collaboration to system deficiencies, organizational structures, or lack of willingness to work in a team [19]. Lack of interprofessional collaboration may be a reality for students in their future clinical practice, and they should be aware of the consequences. In Sweden, interprofessional collaboration in primary care with regard to leg ulcers is often rather limited [17]. Exploring current professional practice in the students’ local context can provide a pedagogical value in discussing even deficits in relation to an ideal picture.

Bateman et al. identified in VP design the VP construction perceived by medical students*,* which included the sub-category Clinical properties, which refers to how VPs can relate to actual clinical practice in “real life” and the importance of authenticity [9]. This was reflected in our results, but our study exposed the interprofessional view of nurses and physicians in practice, which provided an enhanced viewpoint in our VPs from the two professions, and not only the views of medical students. The health professionals’ experience is valuable, and their perspectives can be used to inform further VP authoring in order to enrich VPs for IPE.

We chose the topic leg ulcers for our VPs. Leg ulcers are diagnoses that involve several professions, such as physicians and nurses, and can also include physiotherapists, occupational therapists and social workers. Leg ulcers can therefore involve students from several healthcare programmes and be a well-functioning topic illustrating the importance of interprofessional collaboration. At present, no model or protocol has been presented for assessing the authenticity of clinical practice as represented in VPs. Our study constitutes a step forward towards such a model. We therefore believe our approach can be used to enhance the authenticity of VPs in health professions education and recommend others to perform similar observations in order to improve the educational value of VPs.

### Limitations

The observations and interviews were few, thereby no far-reaching conclusions can be drawn on the themes in more general settings. The risk with observation is that the nurses and physicians can be influenced by the situation of observation and do not behave normally. The person conducting the clinical observation can also be struck with “field blindness” and only observe what he finds interesting. The observations were carried out by a medical student (NK), who has also studied nursing and could therefore be aware of both the nurse’s and the physician’s situation. The interviews were also carried out by a medical student (NK) with limited experience of leg ulcers and interviewing. However, this could be a strength, as the participants who were interviewed might have felt more comfortable and less defensive, as the student was not influenced by a preconceived attitude. No nurse was involved with analysing the material. This was counterbalanced by the three authors having different backgrounds: a medical student who had previously studied nursing (NK), a senior consultant in dermatology (DWE) and a healthcare profession educator and researcher (SE), which thereby increased the possibility of understanding and interpreting the data in multiple ways. Finally, the absence of interviews with patients was a limitation. It would also have been interesting to involve patients and record their views as to what a VP should cover, thereby enriching the cases.

## Conclusions

Authoring VPs risks being reductionistic and reflecting only one professional perspective, which can hamper IPE. The clinical authenticity can be enhanced by using interviews and observations in clinical practice and emphasizing what aspects might be missing. Interviews and observations can highlight the challenges the students may encounter in clinical care of the patient and in the organisation with respect to interprofessional collaboration.

## Supplementary Information

**Additional file 1.**

## Data Availability

The datasets used and analysed during the current study are available from the corresponding author on reasonable request.

## Abbreviations

IPE: Interprofessional education
VP: Virtual patient
