# Supplementary material for: Using interviews and observations in clinical practice to enhance authenticity in virtual patients for interprofessional education
Source: BMC Med Educ. 2020 Nov 25;20:467. doi: 10.1186/s12909-020-02379-9 (PMC7687752; doi:10.1186/s12909-020-02379-9)
Supplement: Supplementary file 1 — Additional file 1. [file 12909_2020_2379_MOESM1_ESM.docx]

# Interview guide

What should a virtual patient with leg ulcer cover, in your view?

Why?

**Physician**

- Patient’s medical history?
- Blood samples/ bacterial swaps?
- Investigations?
- Interventions?

**Nurse**

- Patient’s medical history?
- Nutrition?
- Pain?
- Home situation? Everyday situation?
- Investigations?

**All**

What is it important in interprofessional collaboration?

- What facilitates?
- What complicates?

What can stimulate to increased interprofessional collaboration?

How can the topic of leg ulcers be more interesting to students?

## Additional questions if the person also teach

What do you think is important to focus on?
